# Vegetable Oil-Based Thiol-Ene/Thiol-Epoxy Resins for Laser Direct Writing 3D Micro-/Nano-Lithography

**DOI:** 10.3390/polym13060872

**Published:** 2021-03-12

**Authors:** Sigita Grauzeliene, Aukse Navaruckiene, Edvinas Skliutas, Mangirdas Malinauskas, Angels Serra, Jolita Ostrauskaite

**Affiliations:** 1 Department of Polymer Chemistry and Technology, Kaunas University of Technology, Radvilenu Rd. 19, LT-50254 Kaunas, Lithuania; sigita.grauzeliene@ktu.lt (S.G.); aukse.navaruckiene@ktu.lt (A.N.); 2 Laser Research Center, Faculty of Physics, Vilnius University, Sauletekis Ave. 10, LT-10223 Vilnius, Lithuania; edvinas.skliutas@ff.vu.lt (E.S.); mangirdas.malinauskas@ff.vu.lt (M.M.); 3 Department of Analytical and Organic Chemistry, Universitat Rovira i Virgili, C/Marcel·lí Domingo s/n, Edifici N4, 43007 Tarragona, Spain; angels.serra@urv.cat

**Keywords:** dual curing, optical 3D printing, laser direct writing, click reactions, thiol-ene, thiol-epoxy, linseed oil, soybean oil, biobased polymer

## Abstract

The use of renewable sources for optical 3D printing instead of petroleum-based materials is increasingly growing. Combinations of photo- and thermal polymerization in dual curing processes can enhance the thermal and mechanical properties of the synthesized thermosets. Consequently, thiol-ene/thiol-epoxy polymers were obtained by combining UV and thermal curing of acrylated epoxidized soybean oil and epoxidized linseed oil with thiols, benzene-1,3-dithiol and pentaerythritol tetra(3-mercaptopropionate). Thiol-epoxy reaction was studied by calorimetry. The changes of rheological properties were examined during UV, thermal and dual curing to select the most suitable formulations for laser direct writing (LDW). The obtained polymers were characterized by dynamic-mechanical thermal analysis, thermogravimetry, and mechanical testing. The selected dual curable mixture was tested in LDW 3D lithography for validating its potential in optical micro- and nano-additive manufacturing. The obtained results demonstrated the suitability of epoxidized linseed oil as a biobased alternative to bisphenol A diglycidyl ether in thiol-epoxy thermal curing reactions. Dual cured thermosets showed higher rigidity, tensile strength, and Young’s modulus values compared with UV-cured thiol-ene polymers and the highest thermal stability from all prepared polymers. LDW results proved their suitability for high resolution 3D printing—individual features reaching an unprecedented 100 nm for plant-based materials. Finally, the biobased resin was tested for thermal post-treatment and 50% feature downscaling was achieved.

## 1. Introduction

Laser direct writing (LDW) is a progressive 3D printing technology, which enables production of three-dimensional micro- and nanostructures with variable architectures [1]. LDW is widely applied in polymer additive manufacturing due to its flexibility [2] and precise spatial and lateral resolution [3]. The technique is already routinely used in micro-optics, microelectronics, as well as biomedicine for the polymer-based device fabrication [4]. In polymer additive manufacturing, (meth)acrylate and epoxy monomers has been widely used for optical 3D printing (O3DP) [5]. A promising way to enhance the thermal-mechanical properties of polymers is to combine similar or different stimuli such as temperature or UV light in dual curing process [6,7]. Thus, the dual curing process could be adapted for O3DP technologies. Recently, thermal post curing of acrylate resins was tested in LDW, which was found to facilitate the high property reproducibility that is essential for any application [8]. Additionally, dual curing stereolithographic resins from petroleum-based acrylate and epoxy monomers were formulated and presented as suitable for producing materials with different shape and size [9,10,11]. This area is still largely unexplored as limited amount of publications have been published so far [12].

Click reactions have been used in polymer science due to high reaction rates, oxygen and water insensitiveness, possibility to be initiated either thermally or photochemically [13]. Thiol-click reactions, including thiol-ene and thiol-epoxy click reactions, are the most notable in the last years for preparation of linear, branched and crosslinked polymers [14]. In thiol-ene click reactions of acrylates, not only step-growth thiol-ene copolymerization, but also chain-growth homopolymerization of acrylate can occur, consequently thiols remain partially unreacted [6]. This performance under stoichiometric reaction conditions may help to increase the cross-linking density, glass transition temperature and mechanical properties of the resulting polymers. Additionally, unreacted groups can be controlled on purpose to get partially cross-linked polymer, or second polymerization reaction can be applied to get fully cured material [7]. Thiol-ene cross-linked polymers have been used in adhesives, coatings, biomedical, and electronic packaging materials [15]. Usually, thiol-ene polymers are prepared from petroleum-based materials, such as bisphenol A diallyl ether, diallyl bisphenol A or bisphenol S diallyl ether [16]. The starting compound bisphenol A is used in the manufacturing of plastic food and paper consumer products [17]. However, bisphenol A is considered to cause endocrine, metabolic diseases [18,19] and pollutes water [20]. Therefore, researchers are interested in the replacement of bisphenol by monomers obtained from renewable resources. Environmentally friendly materials, such as isosorbide [21], eugenol [22,23], lignin [24], vanillin [25,26], and vegetable oils [27,28,29] have been already used for thiol-ene click reactions.

Thiol-epoxy click reaction has been widely used for the preparation of adhesives, high-performance coatings, composites [30], hydrogels [31], and shape memory materials [32]. The curing of epoxy resins with thiols is attractive due to the high yield and good mechanical properties of the obtained polymers [33]. The adequate curing temperatures can be achieved by using basic catalysts [34], such as 1-methylimidazole [35], showing no activity under normal conditions and becoming active through external stimulation [36]. About 75% of epoxy resins production in the current market belongs to bisphenol A diglycidyl ether (DGEBA) [37]. Researchers are interested in the replacement of DGEBA by epoxides obtained from renewable resources. Natural phenolic compounds such as tannins [38,39,40], terpenes, cardanols [41], vanillin [42], eugenol [43,44,45,46], and phloroglucinol [47] are commonly used as a substitute for DGEBA due to their rigid structures [48]. Additionally, one of the alternative options is vegetable oils as they are cheap, available in large quantities, and easily modifiable [37]. Ester groups and double bonds can be chemically modified and new functional groups can be introduced in order to prepare polymers with a broad range of properties, e.g., flexibility, adhesion, resistance to water and chemicals [49]. Vegetable oils are also attractive for polymer synthesis due to their low toxicity and higher biodegradability [50,51]. However, long aliphatic chains lead to an excessive flexibility and too low glass transition temperatures [52]. For this reason, aromatic compounds could be used as comonomers for the preparation of polymers from natural oils due to their stability and toughness [53]. Epoxidized oils are also used as substitutes or modifiers for DGEBA [54,55,56,57], but there is still little information about the replacement of DGEBA in thiol-epoxy reactions. Epoxidized linseed, soybean, and olive oils were only incorporated into thiol-epoxy networks where DGEBA was also used [58].

**Figure 1:**
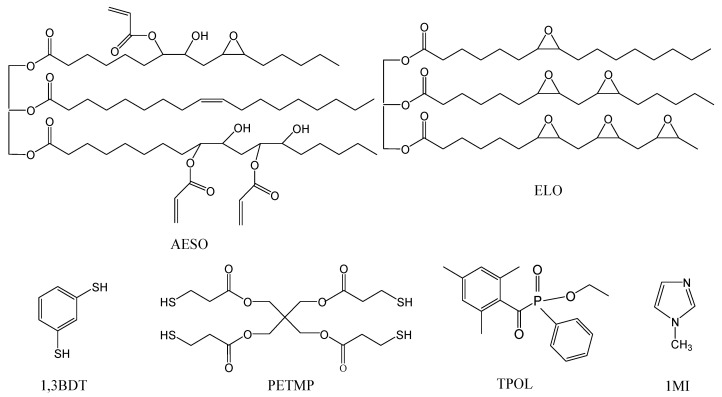
Chemical structures of acrylated epoxidized soybean oil (AESO), epoxidized linseed oil (ELO), benzene-1,3-dithiol (1,3BDT), pentaerythritol tetra(3-mercaptopropionate) (PETMP), ethyl (2,4,6-thimethylbenzoyl) phenyl phosphinate (TPOL), and 1-methylimidazole (1MI).

After considering health and pollution issues of bisphenol A, advantages of vegetable oils, click reactions, and dual curing, the preparation of vegetable oil-based thiol-ene/thiol-epoxy resins that could be used in LDW was chosen. In this study, acrylated epoxidized soybean oil (AESO) and epoxidized linseed oil (ELO) were selected as biobased monomers for thiol-ene, thiol-epoxy, and dual curing with commercially available thiols, benzene-1,3-dithiol (1,3BDT) and pentaerythritol tetra(3-mercaptopropionate) (PETMP) (Figure 1). Non-stoichiometric thiol-ene reactions were chosen to form polymers with the higher amount of the flexible thioether bonds and thus to obtain more ductile and less brittle polymers. Curing kinetics of thiol-ene, thiol-epoxy, and dual curing processes with ethyl (2,4,6-thimethylbenzoyl) phenyl phosphinate (TPOL) as photoinitiator and 1-methylimidazole (1MI) as catalyst were examined by rheological tests. Thermomechanical, thermal stability studies, and mechanical testing of the resulting polymers were carried out. Furthermore, two thiol-ene/thiol-epoxy dual curable formulations were investigated for LDW and evaluated for their applicability as materials for the micro- and nano-scale resolution 3D rapid prototyping. A thermal treatment was performed in order to test their sintering applicability.

## 2. Materials and Methods

### 2.1. Materials

Acrylated epoxidized soybean oil (AESO, an average number of acryloyl groups per molecule 2.7 and 0.3 of epoxide groups), benzene-1,3-dithiol (1,3BDT), pentaerythritol tetra(3-mercaptopropionate) (PETMP), 1-methylimidazole (1MI), and 4-methyl-2-pentanone were purchased from Sigma-Aldrich (Darmstadt, Germany). Epoxidized linseed oil (ELO, having an average number of 6 epoxy groups per molecule) was purchased from Chemical Point (Oberhaching, Germany). Photoinitiator ethyl(2,4,6-thimethylbenzoyl) phenyl phosphinate (TPOL) was purchased from Fluorochem (Hadfield, Derbyshire, UK). All materials were used as received.

### 2.2. Rheometry

Rheological characterization tests were carried out with different thiol-ene and thiol-epoxy resins (Table 1). The mixtures of AESO and 1,3BDT or PETMP (thiol-ene resins, AESO with 1,3BDT named as 100A and AESO with PETMP named as 100C), as well as the mixtures of ELO and 1,3BDT or PETMP (thiol-epoxy resins, ELO with 1,3BDT named as 100B and ELO with PETMP named as 100D) were prepared and mixed together by different ratio (75/25, 50/50 and 25/75 of wt.%). A total of 3 mol.% of TPOL as photoinitiator was used for resins 100A, 100C and their mixtures [59]. In total, 5 phr (parts per hundred of total mixture) of 1MI as catalyst was used for resins 100B, 100D and their mixtures. MCR302 rheometer from Anton Paar (Graz, Austria) equipped with the plate/plate measuring system with Peltier-controlled temperature chamber with the glass plate (diameter of 38 mm) and the top plate PP07 (diameter of 15 mm) was used for rheological measurements. The UV curing procedure of resins 100A and 100C were carried out with shear mode with a frequency of 10 Hz and a strain of 0.3%. The samples were irradiated at room temperature (25.6 ± 2.6 °C) by UV/Vis radiation in a wavelength range of 250–450 nm through the glass plate of the temperature chamber using a UV/Vis spot curing system OmniCure S2000, Lumen Dynamics Group Inc. (Mississauga, ON, Canada). The intensity of the irradiation was 9.3 W cm^−2^ (high pressure 200 W mercury vapor short arc). The thermal curing of resins 100B and 100D was carried out at 150 °C for 1 h with a frequency of 1 Hz and a strain of 1%. Thiol-ene and thiol-epoxy resins (75/25, 50/50 and 25/75 of wt.%) were cured by combining UV and thermal curing. The measuring gap was set to 0.1 mm for all cases. The gel point (*t_gel_*) was determined as the cross-over point of storage (*G*′) and loss (*G*″) modulus.

The viscosity (*η*) of all formulations was measured with a MCR302 rheometer from Anton Paar (Graz, Austria) equipped with a steel parallel plate (top plate diameter of 15 mm) measuring system at room temperature (25 °C). The measuring gap was set to 0.1 mm.

### 2.3. Preparation of Cross-Linked Polymers

Thiol-ene cross-linked polymers named as 100A and 100C were obtained by photopolymerization of AESO with 1,3BDT or PETMP (acryl/SH groups 1:1) using 3 mol.% of TPOL as photoinitiator under a UV lamp (Helios Italquartz, model GR.E 500 W, Milan, Italy) with UV/Vis light at intensity of 310 mW/cm^2^ for 10 min. Thiol-epoxy cross-linked polymers 100B and 100D were obtained by thermal polymerization of ELO with 1,3BDT or PETMP (epoxy/SH groups 1:1) using 5 phr of 1MI as a catalyst. The curing process was carried out at 150 °C for 3 h. Thiol-ene/thiol-epoxy polymers 75A/25B (75 wt.% of thiol-ene mixture of AESO with 1,3BDT and 25 wt.% of thiol-epoxy mixture of ELO with 1,3BDT) and 75C/25D (75 wt.% of thiol-ene of AESO with PETMP and 25 wt.% of thiol-epoxy resin of ELO with PETMP) were prepared by combining UV and thermal curing at 150 °C for 1 h when the sample reached the temperature. All polymers were prepared using 70 mm × 10 mm × 1 mm Teflon molds.

### 2.4. Characterization Techniques

The evolution of thermal curing process of resins 100C and 100D was examined by calorimetric studies on a A Mettler DSC-821 apparatus (Mississauga, ON, Canada). Curing formulations were prepared by mixing ELO and 1,3BDT or PETMP (epoxy/thiol groups 1:1) using 1–5 phr of 1MI as a catalyst. Samples of 10 mg were analyzed under non-isothermal conditions in the temperature range from 30 °C to 250 °C at a heating rate of 10 °C/min under nitrogen atmosphere (nitrogen flow rate 100 mL/min) as described previously [28]. The reaction enthalpy (Δ*h*) was integrated from the calorimetric heat flow signal (*dh/dt*) using a straight baseline with the help of the STARe software.

A Perkin-Elmer Spectrum BX II FT-IR spectrometer (Llanstrisant, UK) was used to record IR spectra of cross-linked polymers. The reflection was measured during the test. The range of wavenumber was (650–4000) cm^−1^.

Dynamic-mechanical thermal analysis (DMTA) was performed in a tensile mode using MCR rheometer from Anton Paar (Graz, Austria). Temperature was ramped from 0 °C to 110 °C at a rate of 2 °C/min. Tests were performed with the samples of the following size: 40 mm × 10 mm × 1 mm. Glass transition temperature (*T_g_*) was defined by the maximum peak of tan*δ* curve. The rubbery modulus (*E*_r_) was determined at *T_g_* + 50 °C from the storage modulus curves.

Cross-linking density (*ν_e_*) of polymers was calculated according to the Flory’s rubber elasticity theory [60]:

(1) $$\nu_{e}=\;\frac{E’}{3\cdot R\cdot T}$$

where *ν_e_* is the cross-linking density (mol/m^3^); *E’* is the apparent rubbery modulus obtained by DMTA from storage modulus curve (Pa); *R* is the universal gas constant (8.314 J/K/mol); *T* is the absolute temperature (K).

Thermal decomposition temperature at the 5% weight loss (*T_dec.−5%_*) and the char yield after thermal degradation of the cross-linked polymers were determined by thermogravimetric analysis (TGA). The measurements were performed on a TA Instruments Q50 apparatus (New Castle, DE, USA) in the temperature range from room temperature to 700 °C at a heating rate of 20 °C/min under N_2_ atmosphere (nitrogen flow rate 100 mL/min).

Mechanical properties of cross-linked polymers were estimated by tensile test on a BDO-FB0.5TH (Zwick/Roell) (Kennesaw, GA, USA) testing machine at 22 °C using the ASTMD-638-V standard. Strain rate of 1 mm/min was used in all cases. Mechanical testing was performed on the dog bone-shaped samples to determine elongation at break, tensile strength, and Young’s modulus. The average values were taken from at least three samples.

LDW 3D lithography was performed employing an ultrafast Pharos laser (515 nm, 300 fs, 200 kHz, Light Conversion Ltd., Vilnius, Lithuania), 63 × NA = 1.4 objective, and combined movements of the linear stages and galvano-scanners. Resolution bridges (RB) method was used to investigate custom-made resin suitability for LDW [61]. The RB model consisted of two rectangle-shaped columns with a width of 25 µm, a length of 60 µm, and a height of 25 µm, which were separated by a gap of 35 µm. The five straight lines were formed in the gaps perpendicularly to the long edges of the columns. Each line was polymerized from a single laser beam scan. RB were obtained with different longitudinal and lateral sizes by varying the laser power (*P*) from 0.01 to 0.15 mW, which corresponded to the light intensity (*I*) at the sample (0.03–0.4 TW/cm^2^), and the scanning velocity (*v*) from 1 to 4 mm/s. Additionally, manufacturing of bulky arc-type objects was presented. It was made of two 25 × 25 µm^2^ columns with a varied height from 5 to 20 µm and distance between the columns from 25 to 100 µm. The columns were connected with a continuous arc on the top. During the fabrication, the resin was placed between two glass slides, creating a layer of 80–100 µm of the resin as was already described previously [62]. After the exposure, the samples were developed in 4-methyl-2-pentanone for 30 min, removing the uncured resin and leaving only the formed structures on the substrate. The fabricated structures were dried with a critical point dryer K850 (Quorum Technologies, East Sussex, UK), sputtered with 10 nm silver layer employing a rotary pumped coater 150R S (Quorum Technologies, East Sussex, UK), and characterized using a scanning electron microscope (SEM, Prisma E, Eindhoven, The Netherlands). For heat treatment, a high temperature hot plate of titanium PZ 28-3T and Programmer PR 5-3T were used (Harry Gestigkeit GmbH, Düsseldorf, Germany). The sample of RB was heated twice: for one hour at 225 °C and later for one hour at 300 °C. After each heat treatment the sample was removed from the hot plate immediately and left in room temperature for couple of minutes to cool down. Then the sample was recoated with a 10 nm silver layer and characterized using SEM.

## 3. Results

### 3.1. Study of Thermal Thiol-Epoxy Curing Process

**Figure 2:**
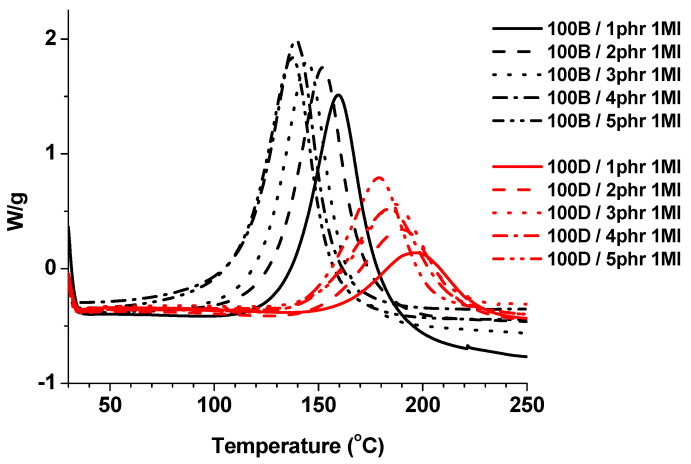
Calorimetric curves corresponding to the curing of ELO with different thiols using 1MI as catalyst.

The thiol-epoxy curing process was examined by calorimetric studies to determine the required amount of catalyst 1MI. The DSC thermograms corresponding to thermal curing of 100B and 100D formulations with 1–5 phr of 1MI are shown in Figure 2. It was determined that 1,3BDT was the more reactive thiol with the lower activation temperature due to the higher acidity of the thiophenols than the thiols and the higher nucleophilicity of the thiophenolate anion compared to PETMP. The reaction of ELO with PETMP started at about 140 °C and was finished at a high temperature, which indicated that the reactivity was rather low. Additionally, the shape of the curves was wider compared to the narrower and higher curves corresponding to the curing of ELO with 1,3BDT, which indicated the faster reaction.

Calorimetric data of the curing of ELO resins with different thiols and 1MI as the catalyst are listed in Table 2. The biggest amount of heat was released using 5 phr of 1MI in the resins of 100B and 100D (443.8 J/g and 256.3 J/g, respectively) leading to the highest curing enthalpy by epoxy equivalent (107.8 kJ/eq and 76.5 kJ/eq, respectively). This indicated that the heat released by epoxy equivalent was higher when the amount of catalyst was increased. According to these results, 5 phr of 1MI was used in the resins of 100B and 100D.

### 3.2. Monitoring Cross-Linking Kinetics by Rheometry

**Figure 3:**
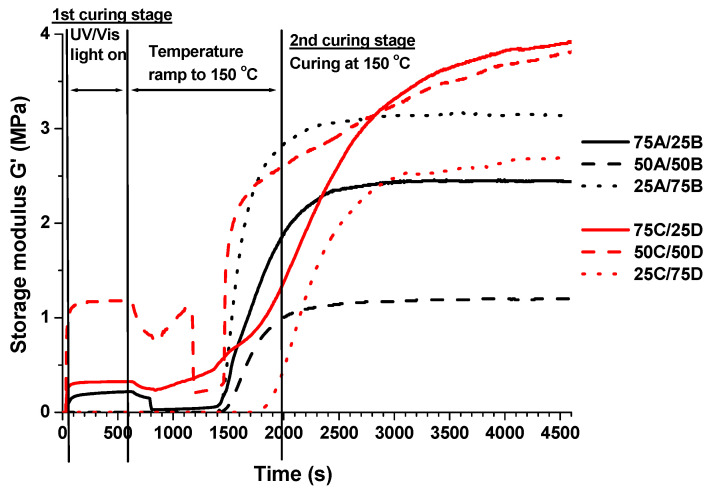
Storage modulus versus curing time of dual cured vegetable oil-based resins.

Rheometry was used to monitor the changes of rheological properties of the resins during UV, thermal and dual curings. Rheometry data of the resins are collected in Table 3. Thiol-ene photocross-linking of the resin 100C containing PETMP as a thiol was faster than that of the resin 100A containing 1,3BDT, as the gel point was reached faster (2 s vs. 3.5 s) and the obtained polymer was more rigid according to the storage modulus (3.79 MPa and 1.56 MPa). The opposite results were obtained with thiol-epoxy thermally cured resins 100B and 100D as thermal polymerization is a slower process compared to photopolymerization. Consequently, by decreasing the amount of thiol-epoxy part in the resin, the gel point was reached faster and rheological characteristics were higher. Storage modulus *G*′ curves versus curing time of the dual cured polymers are shown in Figure 3. During the first curing stage, when UV irradiation was applied, *G*′ of the resins 75A/25B, 75C/25D, and 50C/50D increased, while the other resins remained in liquid form. During intermediate stage when the temperature was ramped to 150 °C, *G*′ of three mentioned polymers started to decrease, indicating glass transition of thiol-ene polymer. After that, *G*′ started to increase, demonstrating the starting of the thiol-epoxy reaction. *G*′ of the other resins: 50A/50B, 25A/75B, and 25C/75D started to increase, after reaching a certain temperature, showing the formation of cross-linked structure by the thiol-epoxy process. During the second curing stage when a constant temperature of 150 °C was applied, *G*′ of all resins increased and reached a plateau. The resins 75A/25B and 75C/25D with the lowest thiol-epoxy part were selected for further investigation of properties, since they were solid after the first curing stage which is essential for LDW and they obtained the highest values of *G*′. The highest viscosity of the resins (Table 1) was also an essential factor for the selection of resins for LDW.

### 3.3. Characterization of Cross-Linked Polymer Structure

**Figure 4:**
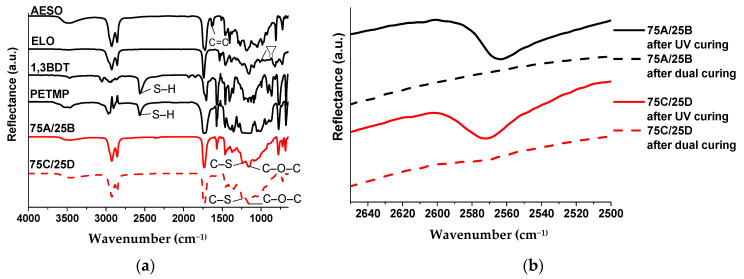
(**a**) FT-IR spectra of AESO, ELO, 1,3BDT, PETMP, cross-linked polymers 75A/25B and 75C/25D; (**b**) FT-IR region corresponding to S–H group absorption signal of the resins 75A/25B and 75C/25D after UV irradiation and after complete curing.

Cross-linked polymers were characterized by FT-IR spectroscopy, which spectra showed the characteristic absorption signals corresponding to their chemical structure (Figure 4). The disappearance of S–H group absorption signal, which was present at 2562 cm^−1^ and 2565 cm^−1^ in the spectra of 1,3BDT and PETMP, was observed in the spectra of the corresponding cross-linked polymers 75A/25B and 75C/25D (Figure 4a). The signal of C=C at 1636 cm^−1^ in the spectrum of AESO and the signal of epoxy group at 940 cm^−1^ in the spectrum of ELO disappeared and the most relevant C–S group stretch at 1233 cm^−1^ appeared in the FT-IR spectra of the cross-linked polymers. In addition, strong absorption signals of C–O–C group at 1151 cm^−1^ and 1154 cm^−1^ were observed in FT-IR spectra of both polymers. Figure 4b shows that the signal of S–H group at about 2570 cm^−1^ is visible after UV irradiation and disappears after thermal curing when polymer is completely cured.

### 3.4. Thermal Characterization of the Materials

Table 4 collects the data obtained by DMTA and TGA analysis. Glass transition temperature (*T_g_*) of photocross-linked thiol-ene polymer 100A (−4 °C) with the aromatic fragments of 1,3BDT was similar to that of the analogous polymer obtained using another photoinitiator (−3 °C) [29]. The *T_g_* of the polymer 100C with the aliphatic fragments of PETMP (1 °C) was also similar to that of the analogous polymer *T_g_* (−1.3 °C) [27]. The thermally cured thiol-epoxy cross-linked polymer 100B with the aromatic fragments of 1,3BDT showed higher *T_g_* than polymer 100D with PETMP fragments (60 °C and 29 °C, respectively). The *T_g_* of polymer 100D (29 °C) was similar to *T_g_* of DGEBA thiol-epoxy polymers with incorporated ELO (25.2–30.4 °C) [58]. The *T_g_* of polymer 100B (60 °C) was similar to *T_g_* of DGEBA and PETMP polymer (59.7–68 °C) [32,63,64].

**Figure 5:**
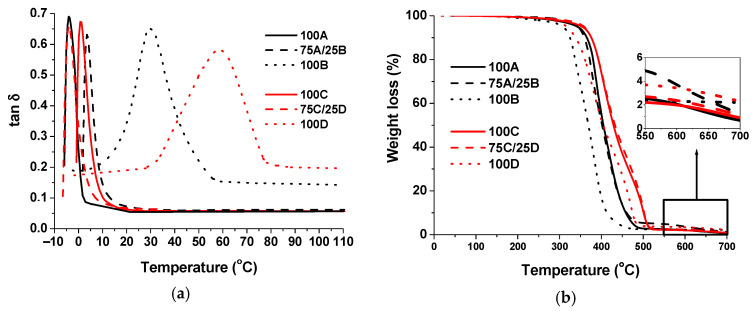
(**a**) Tan*δ* versus temperature curves of the cross-linked polymers; (**b**) Thermogravimetric curves of the cross-linked polymers.

The evolution of tan*δ* versus temperature of the cross-linked polymers with different thiols is shown in Figure 5a. As we can see, the curves of the dual cured polymers 75A/25B and 75C/25D (with maxima at 4 °C and −4 °C) are similar to those obtained by UV cured polymers 100A and 100D (−4 °C, 1 °C). Only thermally cured thiol-epoxy polymers show a displacement to higher *T_g_* and a broadening of the curve. It seems that the lower contribution of the second stage of dual curing (75% UV and 25% thermal) leads to a reduced effect on these characteristics. The highest storage modulus in the rubbery state and the highest cross-linking density correspond to the dual cured polymer 75C/25D (6.38MPa and 768 mol/m^3^) (Table 4). This was probably due to the higher functionality of PETMP compared to 1,3BDT or homopolymerization of AESO that led to the increased degree of cross-linking.

The thermal stability of the polymers prepared was evaluated by TGA. Photocrosslinked thiol-ene polymers 100A and 100C showed higher thermal decomposition temperatures at the 5% weight loss (*T_dec.−5%_*) compared to that of the thermally cured thiol-epoxy polymers 100B and 100D. However, dual cured polymers 75A/25B and 75C/25D showed the highest *T_dec.−5%_* (344 °C and 358 °C, respectively) and intermediary char yield. *T_dec.-5%_* of polymer 100D (317 °C) is similar to *T_dec.−5%_* of DGEBA and PETMP polymer (330–341 °C) [63,64] and is higher than *T_dec.−5%_* of DGEBA thiol-epoxy polymers with incorporated ELO (276–301 °C) [58]. Thermal degradation of polymers with the fragments of 1,3BDT proceeded in one degradation step (Figure 5b). However, the shape of curves of polymers with the fragments of PETMP had a shoulder at higher temperatures due to ester groups of PETMP. It was observed earlier in the TGA curve of the cross-linked polymer of AESO and PETMP [28].

### 3.5. Mechanical Properties

The data of the mechanical characterization of polymers performed on the dog-bone-shaped specimens by tensile test are collected in Table 5. Only polymers obtained by thiol-epoxy curing (100B and 100D) have *T_g_* above room temperature and behave as elastic rigid solid polymers. Polymer 100D might not be fully cured as it has the highest elongation at break value. Polymers with the fragments of 1,3BDT are stiffer, have lower ductility, and brittle behavior, as can be seen from elongation at break, tensile strength and Young’s modulus values, due to the rigid structure of 1,3BDT. Thermally cured thiol-epoxy polymers 100B and 100D showed the highest values of mechanical characteristics compared to UV cured thiol-ene polymers 100A and 100C. Therefore, dual cured polymers 75A/25B and 75C/25D showed higher values compared with polymers 100A and 100C. The tensile strength of ELO-based polymers 100B and 100D (1.44–31.54 MPa) are similar to DGEBA thiol-epoxy polymers with incorporated ELO (8.3–26.4 MPa) [58].

### 3.6. Characterization of 3D Structures Produced by LDW

**Figure 6:**
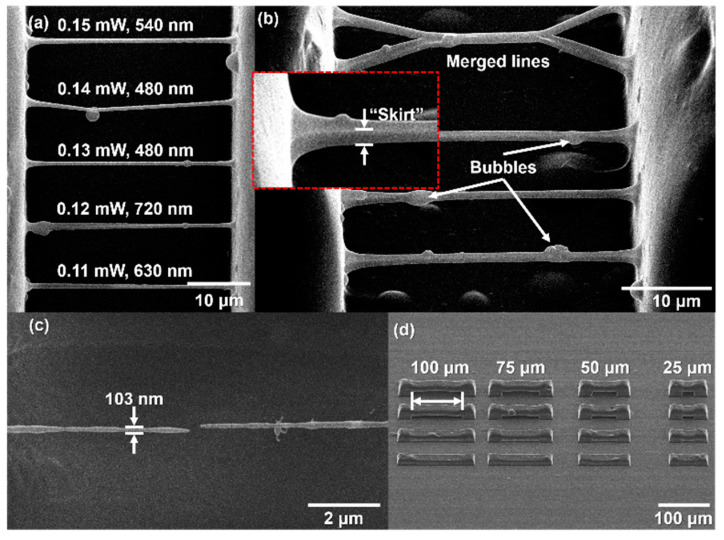
The SEM images of produced objects via laser direct writing (LDW). (**a**) RB manufactured with *P* = 0.11−0.15 mW (*I* = 0.3–0.4 TW/cm^2^) at *v* = 3 mm/s. Averaged line width is depicted in nm; (**b**) RB manufactured with the same *P* but at *v* = 2 mm/s. Common defects are showed: merged lines, formed bubbles and residual material called “skirt”; (**c**) The thinnest observed feature of 103 nm; (**d**) Bulky arc-type objects. Distance between columns is marked with arrowed line and presented in numbers; (**a**,**c**) are top view of the structures, (**b**,**d**) were obtained at 45 degrees angle.

Polymer 75C/25D was used for the 3D micro-/nano-printing experiments as it was less reactive, thus unexposed volume did not fully cure itself during the time needed for the laser writing (which was opposite with polymer 75A/25B). The featured findings are demonstrated in Figure 6. In part (a), RB produced at *v* = 3 mm/s with *P* = 0.11–0.15 mW are showed with the averaged line width. It was possible to achieve a sub-micro feature size, however a tendency to show how it depends on applied exposure was not clearly distinguished as it was influenced by the material itself, its developing and drying conditions. As depicted in the picture, lines manufactured at lower *P* (0.11 and 0.12 mW) were thicker than the ones produced with higher *P* (0.13–0.15 mW), which is a slight increment in exposure dose, yet more sensitive to the individual line formation. These observations can be explained with detailed image of RB at angle of 45 degrees, as shown in Figure 6b. Sometimes two lines might merge to the one making X shape yarn. Often, the thicker bubble-like parts are polymerized within the length of the lines. Lastly, the residual undissolved material, here called “*skirt*” and shown as the close up in a red dashed rectangle, can be detected underneath the main line. Looking from the top, this “skirt” can be seen and measured, making the line appearance thicker than it is. The artificial structure was not designed to be produced and consisted of additional polymerized material stuck to the directly fabricated line. Despite this, such leftovers of the material can be an advantageous result of the laser writing at higher intensity levels, developing process, and drying at the critical point. As presented in Figure 6c, separate low thickness features down to almost 100 nm can be formed, which are self-supported and show no tension even after ablating the line structure while SEM imaging. Here we demonstrate a line of 103 nm thickness produced in biobased resins for the very first time, which is currently a record value for it. Previously reported features were of 500 nm [65] and sub-400 nm [66], respectively. Finally, bulky arc-type objects are displayed in Figure 6d. It reveals that complex architecture structures can be manufactured reproducibly out of polymer 75C/25D, as hanged bulk parts of 100 μm length survive even without critical point drying. In summary, the developed plant-based resin was validated for its potential usage in LDW 3D micro-/nano-lithography, individual features can reach an unprecedented 100 nm dimensions, yet some further efforts are needed for optimising material homogeneity to fit industrial demands.

**Figure 7:**
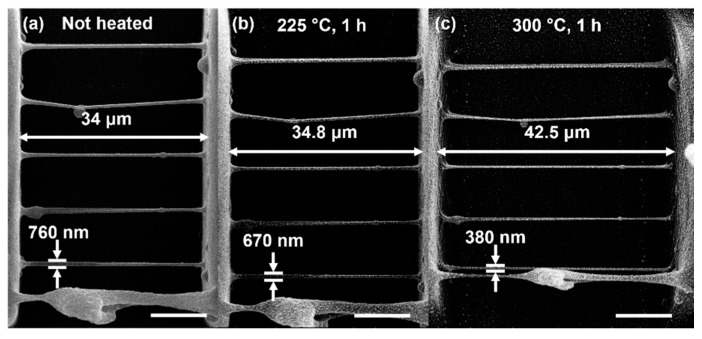
The SEM images of the same RB structure and applied thermal treatment in ambient surrounding: (**a**) before, (**b**) after 1h at 225 °C, (**c**) after additional 1h at 300 °C. It is seen that the structures preserve their shape while downscaling 12% and 50% of their initial dimensions (the line thickness). Scale bar in all images represents 10 μm.

Finally, the fine RB structures were tested for thermal post-processing and proved its potential for the downscaling by up to 50% via pyrolysis, which is a known technique for hybrid materials [67,68,69], yet have not been applied to plant-based bioresins [70]. In Figure 7b,c, the line thickness was decreased, possibly due to several reasons: due to the polymer thermal decomposition which started at such high temperatures and due to the necking of the line due to its plastic deformation caused by the tensile stress that occurred as the distance between the line supports increasing. The line does not break due to the elongation of the flexible chains present in the polymer. The formed stable neck describes polymer 75C/25D as a ductile polymer suitable for thermal post-processing via pyrolysis which is a powerful method for further downscaling the micro-/nano-objects and densifying the material for optimizing the functional performance of the 3D structures.

## 4. Conclusions

Vegetable-oil-based thiol-ene/thiol-epoxy polymers were synthesized from acrylated epoxidized soybean oil, epoxidized linseed oil and thiols (benzene-1,3-dithiol and pentaerythritol tetra(3-mercaptopropionate)) using ethyl(2,4,6-thimethylbenzoyl) phenyl phosphinate as the photoinitiator and 1-methylimidazole as the catalyst. Benzene-1,3-dithiol was the most reactive thiol with the lowest activation temperature due to the higher nucleophilicity of thiophenolate anion compared to the thiolate from pentaerythritol tetra(3-mercaptopropionate). The use of benzene-1,3-dithiol for thiol/epoxy reaction led to the cross-linked polymers with the higher glass transition temperature, stress at break, and Young’s modulus due to the more rigid structure. Dynamic-mechanical thermal analysis, thermogravimetry, and mechanical testing of thiol-epoxy polymers confirmed that epoxidized linseed oil can be a good biobased alternative to bisphenol A diglycidyl ether in thiol-epoxy thermal curing reactions. Rheometry and mechanical testing showed that dual cured polymers exhibited higher rigidity and higher tensile strength, and Young’s modulus values compared with UV-cured thiol-ene polymers. Femtosecond LDW lithography was employed for validating the applicability of biobased thiol-ene/thiol-epoxy resin with pentaerythritol tetra(3-mercaptopropionate) for high resolution O3DP. Namely, the possibility was demonstrated to perform arc-type microstructures in a reproducible manner and 100 nm individual features were obtained by optimizing the exposure and developing conditions. Additionally, the structures were heated up to 300 °C and isotropic shrinkage was achieved. The results show a high potential of the vegetable-oil-based resins for high fidelity rapid prototyping and additive manufacturing.

## Figures and Tables

**Table 1:** Composition and viscosity of prepared formulations.

Resin *	Thiol	Amount of AESO, (wt.%)	Amount of ELO, (wt.%)	Amount of thiol, (wt.%)	Amount of TPOL, (wt.%)	Amount of 1MI, (wt.%)	Viscosity *η*, Pa·s
100A	1,3BDT	88.16	0	10.53	1.31	0	17.26 ± 0.40
75A/25B		65.56	17.03	15.26	0.98	1.17	13.32 ± 0.23
50A/50B		43.26	33.75	19.98	0.58	2.43	3.71 ± 0.69
25A/75B		21.43	50.16	24.51	0.29	3.61	0.54 ± 0.01
100B		0	66.41	29.01	0	4.58	0.12 ± 0.01
100C	PETMP	81.57	0	17.43	1.00	0	10.06 ± 0.02
75C/25D		60.61	13.93	23.54	0.75	1.17	4.03 ± 0.02
50C/50D		40.00	27.67	29.42	0.49	2.42	2.10 ± 0.09
25C/75D		19.80	41.09	35.24	0.25	3.62	1.90 ± 0.10
100D		0	54.20	41.22	0	4.58	0.55 ± 0.02

* A—thiol-ene resin of AESO with 1,3BDT; B—thiol-epoxy resin of ELO with 1,3BDT; C—thiol-ene resin of AESO with PETMP; D—thiol-epoxy resin of ELO with PETMP; 100, 75, 50, 25—amount (wt.%) of thiol-ene (A or C) and/or thiol-epoxy (B or D) resin in the mixture.

**Table 2:** Calorimetric data of the curing of epoxidized linseed oil (ELO) with benzene-1,3-dithiol (1,3BDT) and pentaerytritol tetrakis (3-mercaptopropionate) (PETMP) containing different amounts of 1-methylimidazole (1MI).

Resin	Proportion of 1MI (phr ^1^)	∆*h* ^2^ (J/g)	∆*h* ^3^ (kJ/eq)
100B	1	400.1	93.5
	2	405.6	95.8
	3	409.3	97.5
	4	427.4	102.9
	5	443.8	107.8
100D	1	139.4	39.7
	2	190.0	57.1
	3	207.0	60.6
	4	225.6	66.7
	5	256.3	76.5

^1^ phr—parts per hundred of the monomer mixture; ^2^ curing enthalpy measured in a DSC scan at 10 ^°^C/min; ^3^ curing enthalpy by epoxy equivalent of the initial mixture.

**Table 3:** Rheological characteristics of vegetable oil-based thiol-ene/thiol-epoxy mixtures.

Resin	Storage Modulus *G*′ (MPa)	Loss Modulus *G*″ (kPa)	Complex Viscosity η* (MPa·s)	Gel Point *t*_gel_ (s)
100A	1.56 ± 0.05	257.72 ± 27.04	25.15 ± 0.85	3.5 ± 0.5
75A/25B	2.44 ± 0.00	10.25 ± 1.38	38.85 ± 0.01	7.0 ± 0.0
50A/50B	1.30 ± 0.10	2.98 ± 0.09	20.55 ± 1.45	1340.1 ± 35.7
25A/75B	3.15 ± 0.00	60.58 ± 0.00	50.10 ± 0.00	1390.2 ± 0.0
100B	5.19 ± 0.60	106.77 ± 0.61	82.55 ± 9.55	786.6 ± 15.6
100C	3.79 ± 0.30	1415 ± 385	64.50 ± 6.60	2.0 ± 0.0
75C/25D	3.96 ± 0.00	12.56 ± 0.00	63.03 ± 0.00	2.0 ± 0.0
50C/50D	3.87 ± 0.00	11.37 ± 0.00	61.62 ± 0.00	2.0 ± 0.0
25C/75D	2.70 ± 0.00	9.94 ± 0.00	42.90 ± 0.00	1692.6 ± 0.0
100D	3.36 ± 0.29	25.80 ± 0.00	53.35 ± 4.55	1170.0 ± 0.0

**Table 4:** Dynamic mechanical thermal analysis (DMTA) and thermogravimetric analysis (TGA) data.

Polymer	DMTA			TGA	
	*T_g_*^1^ (°C)	*E_r_*^2^ (MPa)	*ν_e_*^3^ (mol/m^3^)	*T_dec.−5%_*^4^ (°C)	Char yield ^5^ (%)
100A	−4	3.98	479	343	0.7
75A/25B	4	5.10	614	344	1.3
100B	60	6.16	742	308	2.2
100C	1	5.64	679	354	0.9
75C/25D	−4	6.38	768	358	1.1
100D	29	3.92	472	317	2.4

^1^ Temperature of the maximum of tan*δ* estimated by DMTA; ^2^ Storage modulus in the rubbery state determined at *T_g_* + 50 °C; ^3^ Cross-linking density calculated according to the Flory’s rubber elasticity theory; ^4^ Temperature at the weight loss of 5% obtained from TGA curve; ^5^ After thermal degradation in N_2_ atmosphere.

**Table 5:** Mechanical characteristics of polymers.

Polymer	Elongation at Break (%)	Tensile Strength (MPa)	Young’s Modulus (MPa)
100A	5.75 ± 0.23	0.65 ± 0.03	9.53 ± 4.42
75A/25B	3.26 ± 0.03	0.91 ± 0.09	13.46 ± 1.81
100B	1.92 ± 0.06	31.54 ± 2.72	2458 ± 43.90
100C	1.95 ± 0.82	0.82 ± 0.50	4.00 ± 2.09
75C/25D	4.97 ± 1.11	0.87 ± 0.01	8.76 ± 2.22
100D	7.03 ± 1.05	1.44 ± 0.43	23.3 ± 0.96

## Data Availability

The data presented in this study are available on request from the corresponding author.
